# Examination of conversion method of dose distribution of lung cancer IMRT using fluence reversible calculation function in O-ring type linac and C-type linac

**DOI:** 10.1007/s13246-022-01122-6

**Published:** 2022-04-19

**Authors:** Toshiya Rachi, Raturi Vijay Parshuram, Yuki Tanaka, Haruki Togo

**Affiliations:** 1 Department of Radiological Technology, National Cancer Center Hospital East, 6-5-1, Kashiwanoha, Kashiwa, Chiba 277-8577 Japan; 2 Department of Radiation Oncology and Particle Therapy, National Cancer Center Hospital East, Kashiwa, Japan; 3 Course of Advance Clinical Research of Cancer, Graduate School of Medicine, Juntendo University, Bunkyo, Japan

**Keywords:** O-Ring Type Linac, C-Type Linac, Fluence Reversible Calculation, Lung Cancer

## Abstract

Generally, converting irradiation plans between C-arm linacs (C-linac) when the linac fails is possible without recalculating the dose distribution using a treatment planning system (TPS), because they have similar mechanical structure. However, the O-ring-type linac (O-linac) differs from the C-linac in forming the dose distribution. Therefore, if O-linac breaks down, it is necessary to formulate a treatment plan from scratch. In this study, we investigated a method for converting irradiation from an O-linac to a C-linac. Thirty patients with lung cancer who underwent volumetric-modulated arc therapy with an O-linac were included in this study. The O-linac dose distribution was converted into energy fluence by the function of the TPS. The alternative linac multi-leaf collimator (MLC) was then optimized to achieve energy fluence. The homogeneity index, conformity index, and planning treatment volume (D95%, D2%) of the converted plan were compared with the original plan. For organ at risk (OAR), the dose-volume histograms (DVHs) of the lung, esophagus, heart, and spinal cord were evaluated. Additionally, the shapes of the isodose curves were compared using the Dice similarity coefficient (DSC). There was no significant difference between the target and OARs (p > 0.05). The mean DSCs of 30% to 100% isodose curves of the prescribed dose and the isodose ≥ 105% and ≤ 20%were > 0.8 and < 0.8, respectively. Due to the structural differences of MLC, the dose-volume and generation positions were different in the dose range of ≥ 105% and ≤ 20%; hence, DSCs decreased. However, no statistically significant difference in the DVH was identified for either treatment plan. Based on this result, we propose a simple replanning method for performing MLC fitting after converting the dose to the energy fluence.

## Introduction

Varian Medical Systems operates an O-ring-type linear accelerator (linac) called a Halcyon. Halcyon has a dual-layer multi-leaf collimator (MLC) that is flattening filter-free (FFF) and has a 6X-FFF single beam only specification. It is possible to perform three-dimensional conformal irradiation and intensity-modulated radiation therapy (IMRT) [1].

Generally, converting irradiation plans between C-arm linacs (C-linac) when the linac fails is possible without recalculating the dose distribution using a treatment planning system (TPS), because they have similar mechanical structure. However, because the mechanical structure of Halcyon has an FFF, the process for forming the dose distribution is different from that of the C-linac [2]. Therefore, if Halcyon becomes unusable owing to a breakdown or inspection, it will be necessary to formulate a treatment plan from scratch, which will take a significant amount of time. Furthermore, in such situation, radiation cannot be immediately delivered to the patient. Therefore, it is of great benefit to patients to clarify the consistency of treatment plans between these linacs and to establish a more straightforward converted plan creation method.

There are many reports comparing Halcyon and C-linac. Li et al. reported the difference in quality control from C-linac [3], and OGrady et al. compared the characteristics and safety of the dose distribution of each linac [4]. In a report similar to this study, Li et al. compared the dose distribution of IMRT created by C-linac on TPS with that produced by Halcyon^2^. To date, no specific method for performing converted irradiation has been reported. Proposals for reshaping treatment plans are considered globally beneficial. In this study, we investigated a method for converting Halcyon to C-linac irradiation.

## Material and methods

### Treatment plans and target patients

RayStation version 9.0.0 (RaySearch Laboratories, Stockholm, Sweden) and Eclipse version 13.6.8 (Varian Medical System, Inc., Palo Alto, CA) TPS were used. A plan for 30 lung cancer cases was created using a Halcyon 6X-FFF beam on Eclipse. Lung cancer cases included in our study were histopathologically confirmed as small-cell lung cancer, non-small cell lung cancer, and stage III cancer according to the Unio Internationalis Contra Cancrum 8th edition [5]. The prescribed doses ranged from 45 to 60 Gy.

All volumetric-modulated arc therapy plans on Halcyon were created with two or three arcs using a 6X-FFF. The dose constraint index for all plans was based on guidelines proposed by the Japanese Society for Radiation Oncology [6]. The optimal plan was approved when at least ≥ 95% of planning treatment volume (PTV) received 100% of the dose. For the lung, constraints were V20 Gy ≤ 40%, V10 Gy ≤ 40%, V5 Gy ≤ 60%, and mean dose (Dmean) ≤ 20 Gy. For the esophagus, the constraint was V60 Gy ≤ 19 Gy. For the heart, the dose constraint was V50 Gy ≤ 25%. The maximum dose (Dmax) to the spinal cord was limited to ≤ 45 Gy.

### Creating a converted plan

Using the fall back planning (FBP) tool in RayStation, the Halcyon dose distribution was converted to a dose distribution using a 6X beam with a TrueBeam RT system (Varian Medical System, Inc., Palo Alto, CA). The FBP converts the dose data of the original plan into energy fluence. The alternative linac MLC is then optimized to achieve energy fluence. Suppose there are unachieved constraints of the region of interest in the plan converted by the FBP. In this case, the plan is completed by adding the optimization parameters of the corresponding elements and performing the optimization. For all alternative plans, the treatment plan is completed by implementing the FBP tool, or the treatment plan is completed with only one additional optimization. Reoptimization is performed in 10 out of the 30 cases, and it is a simple method of adding objects that do not satisfy the dose constraint. Therefore, there is no need to reconsider the optimization, and it does not depend on the operator’s skill.

**Fig. 1:**
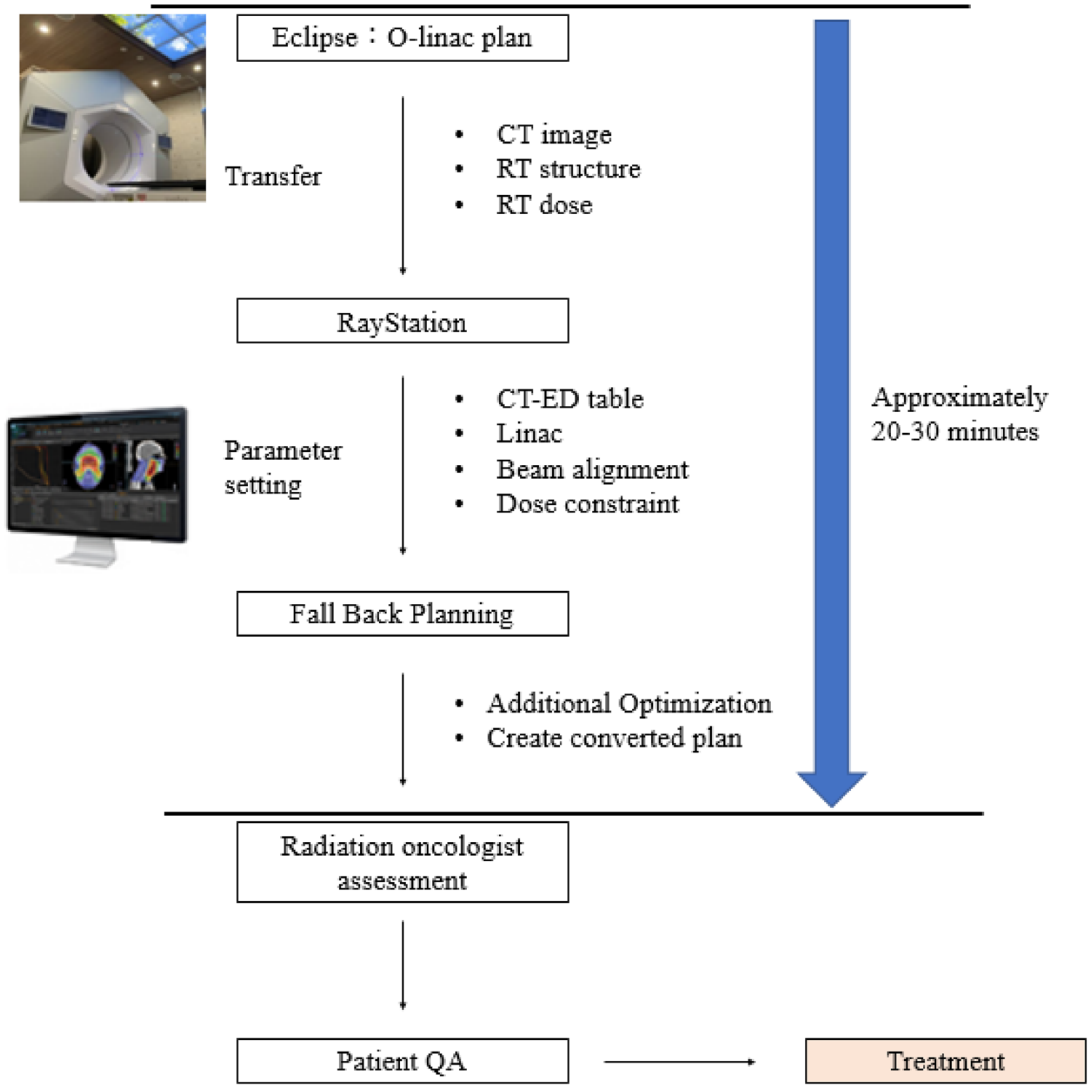
Overall workflow using the fall back planning tool

The completed alternative plan was confirmed by two people: the radiation oncologist who co-authored the study and the radiation oncologist who was in charge of the original plan. The overall workflow of the FBP tool is shown in Fig. 1.

The time required to convert one plan is approximately 20–30 min. If planning is performed from the beginning using the C-linac, it is necessary to reconsider the object value for optimization. Therefore, it generally takes at least 1–3 h to complete the plan.

### Evaluation of the converted plan

The dose constraints of the conversion plan were evaluated using a dose-volume histogram (DVH). The homogeneity index (HI), conformity index (CI), and PTV (D95%, D2%) of the modified plan using TrueBeam 6X were evaluated. For organ at risk (OAR), the DVHs of lung (V5%, V10%, V20%, and Dmean), esophagus (V60%), heart (V50%), and spinal Cord (Dmax) were evaluated. The evaluation confirmed whether there was a difference between the DVHs of the original and the converted plans. The HI and CI are defined as follows:

$$HI = \frac{{D_{max} - D_{min} }}{{D_{pres} }},\,CI = \frac{{\left( {V_{PTV} \cap V_{Pres} } \right)^{2} }}{{V_{PTV} + V_{pres} }}$$

For each plan, the isodose curves for every 10% were compared, and the evaluation index of similarity by the Dice similarity coefficient (DSC) was used. DSC is defined as follows:

$$Dice \ \ similarity \ \ coefficient = \frac{{2\left| {V_{iso\_Hal} \cap V_{iso\_True} } \right|}}{{\left| {V_{iso\_Hal} } \right| + \left| {V_{iso\_True} } \right|}}$$

${V}_{iso\_Hal}$ and ${V}_{iso\_true}$ are the same isodose curves for Halcyon and TrueBeam, respectively. An index of DSC, which is the threshold for similarity (DSC ≥ 0.8), is shown in Task Group 132, published by the American Association of Physicists in Medicine (AAPM) [7]. An objective evaluation is possible using these quantitative indices.

### Patients’ quality assurance (QA)

Patient-specific quality assurance was performed using a Delta4 dosimetry diode array and ionization chamber. The tolerance value in Delta4 was evaluated using γ-index analysis (dose difference, 3%; distance to agreement, 2 mm). The tolerance value of the dose difference in the ionization chamber was within ± 3%. The tolerance value was based on the AAPM TG-218 [8]. Both the original and alternate plans were evaluated based on these tolerances.

## Results

### Dose-volume histogram of the target and organs at risk

**Fig. 2:**
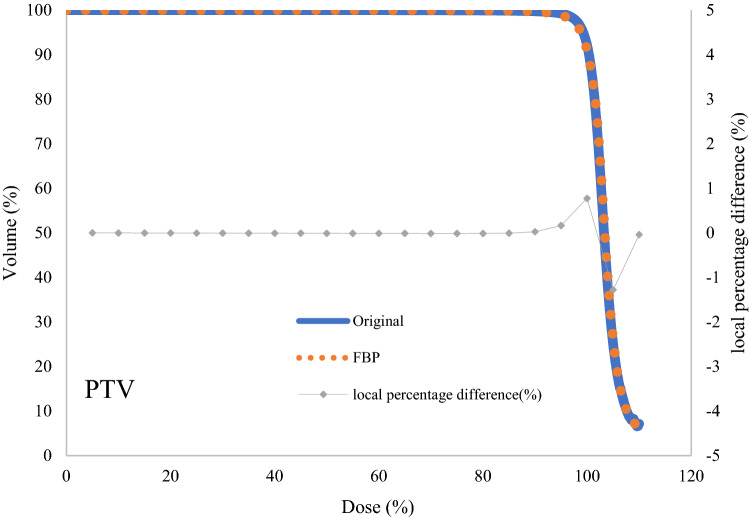
The comparison results of the mean dose-volume histograms of PTV between the original plan on Halcyon and the converted plan on TrueBeam for 30 cases of lung cancer. The local percentage difference is plotted every 5% of the total dose

The comparison results of the mean DVHs of the PTV between the original plans on Halcyon and the FBP plans on the TrueBeam RT system for 30 lung cancer cases are shown in Fig. 2. The statistical results of the DVH metrics for the PTV and OARs are reported in Table 1. There were no significant differences in D2%, D95%, HI, and CI (p > 0.05). The D2% and D95% in Halcyon plans were similar to those of the conversion program, which were 106.04 ± 8.31% vs. 106.34 ± 8.36% (p = 0.889) and 94.92 ± 19.25% vs. 94.61 ± 19.21% (p = 0.951), respectively. The CI and HI of the Halcyon plan and the FBP plan were 1.12 ± 0.05 vs. 1.12 ± 0.05 (p = 0.77) and 1.11 ± 0.16 vs. 1.06 ± 0.19 (p = 0.26), respectively.

**Table 1:** DVH metrics for PTV and OAR

ROI	Parameters	Original	SD	FBP	SD	p_value
PTV	D2%	106.04	8.31	106.34	8.36	0.89
	D95%	94.92	19.25	94.61	19.21	0.95
	HI	1.12	0.05	1.12	0.05	0.77
	CI	1.11	0.16	1.06	0.19	0.26
Lung	V5%	53.74	8.92	54.07	8.52	0.88
	V10%	38.51	7.36	37.88	7.55	0.74
	V20%	23.51	7.43	23.28	7.35	0.90
	Dmean(Gy)	6.29	2.89	6.36	2.61	0.92
Heart	V10%	32.23	20.48	31.91	20.73	0.95
	V20%	22.27	16.64	21.71	16.67	0.90
	V30%	15.14	11.80	14.76	11.10	0.90
	V50(Gy)	4.97	5.33	4.70	5.00	0.84
	Dmean(Gy)	11.54	6.75	11.48	6.72	0.88
Esophagus	V10%	61.41	13.87	60.35	14.76	0.78
	V20%	47.10	18.69	46.16	19.61	0.85
	V30%	37.40	20.98	36.29	21.38	0.84
	V60(Gy)	8.99	14.81	7.58	13.44	0.70
	Dmean(Gy)	24.26	9.33	23.69	9.83	0.82
Spinal cord	Dmax(Gy)	36.22	9.99	35.97	10.64	0.92

**Fig. 3:**
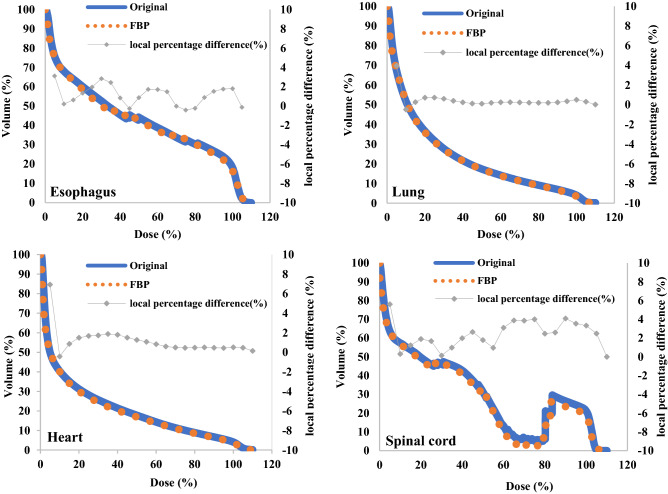
The comparison results of the mean dose-volume histograms of OARs between the original plans on Halcyon and the converted (FBP) plans on TrueBeam for 30 cases of lung cancer. The local percentage difference is plotted every 5% of the total dose

**Fig. 4:**
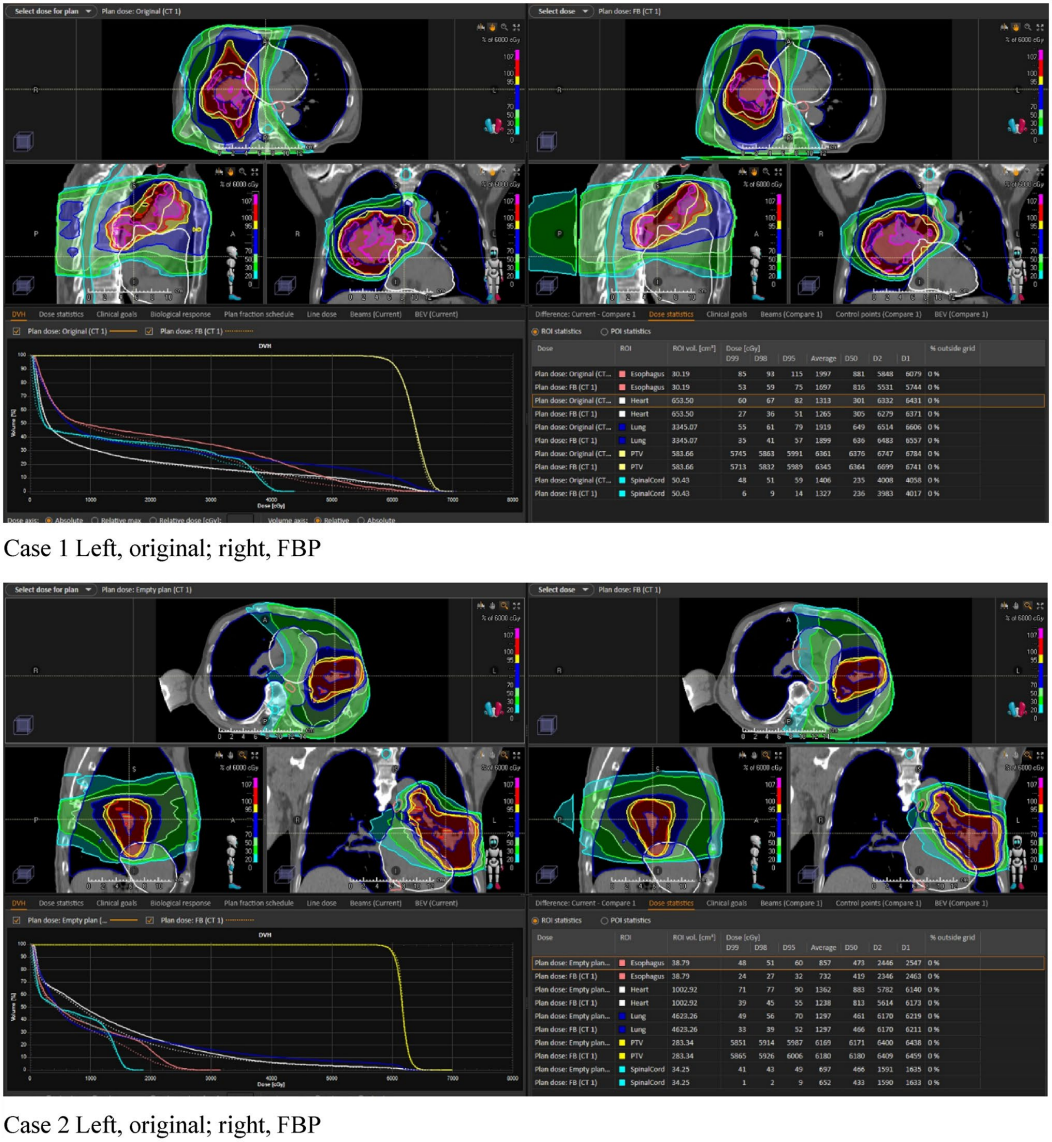
These are two cases of the dose distribution of the original plan and FBP plan on RayStation. There is no big difference in the apparent dose distribution, which shows the convenience of the FBP tool. There was no significant difference in each dose-volume histogram

Lung V5% and Dmean (Gy) were 53.74 ± 8.92% vs. 54.07 ± 8.52% (p = 0.88) and 6.26 ± 2.89 Gy vs 6.36 ± 2.61 Gy (p = 0.92) in the original plan and FBP plan, respectively. These values were slightly higher in the conversion plan, but the difference was not statistically significant (p > 0.05). In contrast, the FBP plans for lung V10 (%) and V20 (%), heart V10 (%), V20 (%), V30 (%), V50 (Gy), Dmean (Gy), esophagus V10 (%), V20 (%), V30 (%), V60 (Gy), Dmean (Gy), and spinal cord Dmax (Gy) were slightly lower than the original plans. However, these differences were not statistically significant (p > 0.05). Figure 3 shows the mean DVH in the lung, heart, esophagus, and spinal cord for all patients. In addition, Fig. 4 shows two cases of dose distribution of the original plan and the FBP plan on RayStation.

### Dice similarity coefficient of the isodose curve

**Fig. 5:**
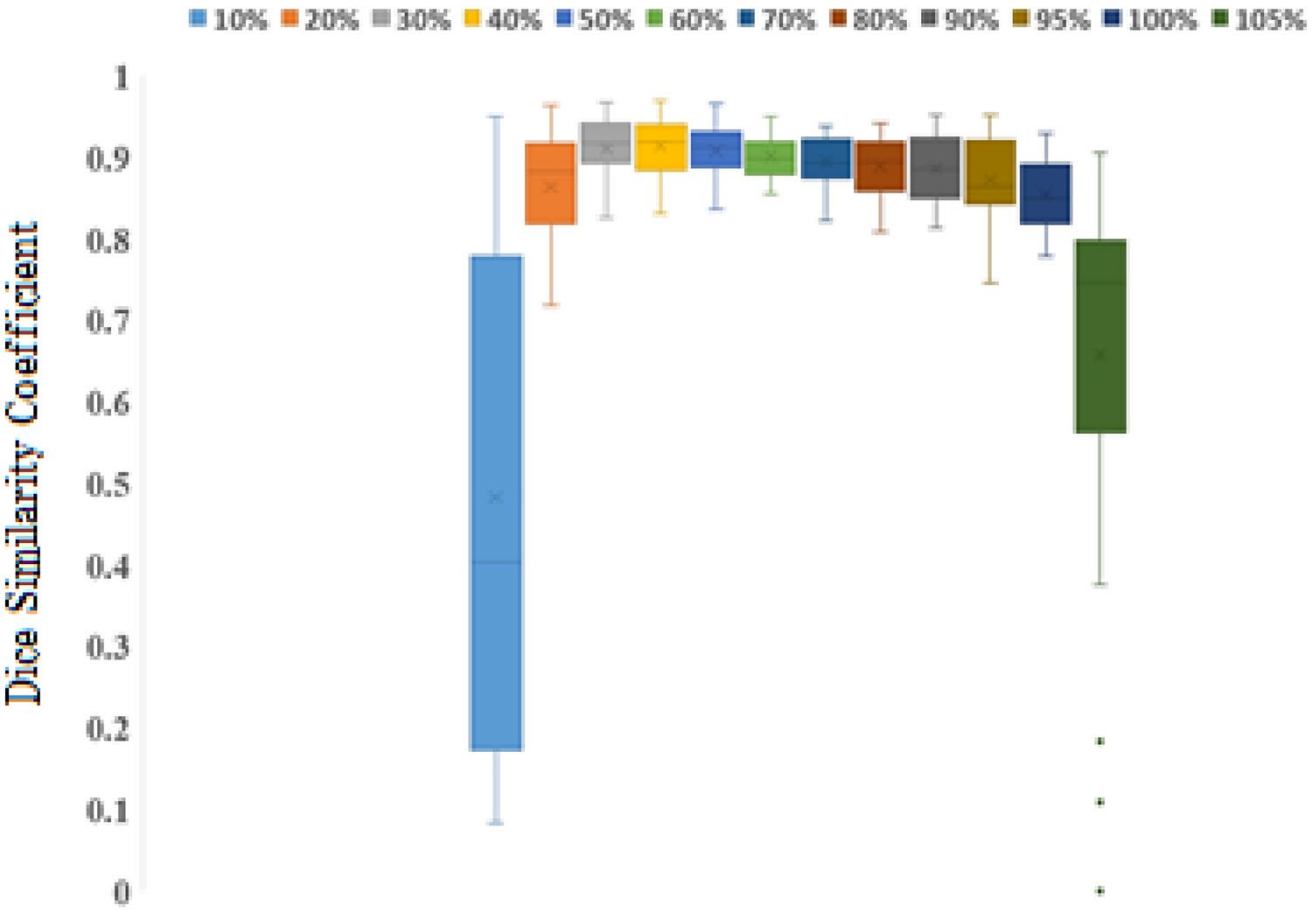
Dice similarity coefficient of the isodose curve of the treatment plan created by Halcyon 6X-FFF and FBP plans by TrueBeam 6X in 30 cases of stage III lung cancer

Figure 5 shows the DSC calculated from each isodose curve for all patients. The mean DSC values are presented in Table 2. In all patients, the 30%–100% isodose curve of the prescribed dose was > 0.8; this is an indicator of the DSC shown on the TG132, and it displays a great match^7^. In contrast, the isodose curve for < 20% was < 0.8. In the high dose range > 105%, the dice coefficient was well below 0.8.

**Table 2:** Mean value of Dice similarity coefficient of the each isodose curve

Isodose level	DSC
10%	0.483 ± 0.306
20%	0.864 ± 0.072
30%	0.911 ± 0.041
40%	0.914 ± 0.037
50%	0.908 ± 0.032
60%	0.901 ± 0.027
70%	0.894 ± 0.029
80%	0.889 ± 0.037
90%	0.887 ± 0.041
95%	0.873 ± 0.050
100%	0.855 ± 0.046
105%	0.658 ± 0.234

### Results of patient QA

**Fig. 6:**
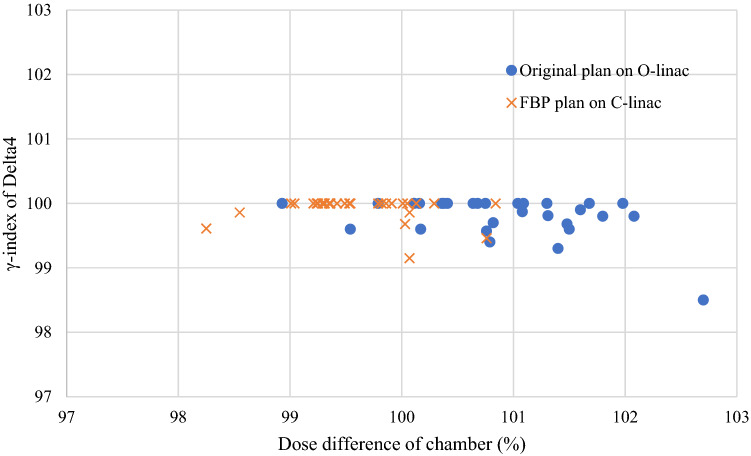
Results of the verification using the Delta4 and the ionization chamber. It was confirmed that all treatment plans of the original and FBP plans were within the tolerance value. This shows the highest match rate when the γ-index is 100 and the dose difference is 100%

The results of the verification using Delta4 showed a high agreement of γ-index of 98% or more in all patients. Moreover, it was verified that the tolerance range of the ionization chamber was ± 3% of the absolute dose. Therefore, it was confirmed that all treatment plans for the original and alternative plans were within tolerance values. The results are shown in Fig. 6.

## Discussion

The Halcyon O-ring linac is used worldwide, and several studies have been published on the safety and quality control of treatment plans made with Halcyon. Li et al. conducted IMRT dose planning for cervical cancer in Halcyon and C-arm linac; the dose distribution in Halcyon showed the same coverage for the target and a reduced dose for OARs compared to C-linac^2^. Halcyon has a faster gantry speed than C-linac and the treatment can be completed in a short time [9]. A similar result was reported by Riley et al. for prostate and head and neck cancers, where IMRT dose planning for Halcyon was compared with C-linac [10]. Based on these reports, Halcyon can deliver radiation equal to or better than C-linac. However, there are no reports on the conversion of the treatment plan created by Halcyon into one using a C-linac. If there is a breakdown of Halcyon, converting the treatment plan created using Halcyon to another C-linac will greatly benefit clinical practice.

This study found no significant differences in CI, HI, PTV, and OARs (lung, heart, esophagus, and spinal cord) between the original plan and conversion plan created using FBP for lung cancer. From this result, it is considered that the original plan for Halcyon and the FBP plan for TrueBeam are equivalent.

DSC was used to calculate the similarity in the shapes of the isodose curves. According to TG-132, the tolerance value of DSC was ≥ 0.8. No significant difference was noted in the shape of the isodose curve between the original and FBP plans in the isodose range of 20–100%. However, the similarity was low in the regions of the isodose curve of ≤ 10% and ≥ 105%. These differences are considered to be largely due to the structural differences between Halcyon and C-linac. Halcyon has a dual-layer MLC with an FFF, and C-linac has a single-layer MLC with a flattening filter. The transmission of MLC is an important factor in IMRT. Li C et al. and Yao W et al. reported that the transmittance of Varian millennium 120 MLC is approximately 1.5% [11, 12]. In contrast, the transmittance of dual-layer MLC in Halcyon has also been reported to be 0.42% by Roover et al. and 0.41% for distal MLC and 0.4% for proximal MLC by Lim et al. [13, 14]. The FFF beam reduces transmission from the MLC [15]. Because of FFF beam characteristics and MLC structural differences, it is deemed that the DSC was greatly reduced due to the difference in the generation position and dose-volume in the high dose region of ≥ 105%.

Vassiliev et al. reported that the FFF beam had a slight change in the deep lateral dose curve and was suitable for protecting normal tissues [16]. Similarly, Halcyon does not have jaws; therefore, less scattered radiation is generated from it. Rovert et al. stated that Halcyon could reduce the dose to normal tissue by reducing the influence of scattered radiation in the lateral direction compared to the C-linac [17]. Because the dose profile of Halcyon is convex, it is not easy to compare and evaluate the quantitative penumbra with the flat profile of C-linac. However, the slight difference in the low dose range was probably caused by the small transmission from the MLC mentioned above and the structure without the jaw. In this study, the Halcyon treatment plan was converted to a treatment plan with two full-arc rotations using the TrueBeam 6X beam. A future development point is to study the optimal values for these parameters in treatment plans using TrueBeam, such as the X-ray energy, beam angle, and number of beams to be converted.

In addition, the uncertainty of the beam modeling of C-linac and O-linac in TPS also affects the dose distribution. In particular, the dosimetric leaf gap (DLG) and MLC transmission are considered to contribute to the difference between the low- and high-dose regions. According to a report by Maloory et al., the effect of DLG on Eclipse and RayStation had a change in target dose from − 5% to + 3% and − 4% to + 7%, respectively, with the same change in offset value. The effect of MLC transmission resulted in changes of up to 1% and 2% [18]. In other words, beam modeling of the planning device causes a slight dose difference.

## Conclusion

It was possible to convert the dose distribution planned on the Halcyon to the dose distribution of the conventional C-arm-type linac. A simple replanning method called FBP, which performs MLC fitting after converting from dose to energy fluence, has been proposed and is an effective means for dealing with breakdowns in an actual treatment site.

RayStation does not support O-linac, such as Halcyon, and a treatment plan from C-linac to O-linac in the opposite direction to this study is expected in the future. Moreover, this study suggests that the FBP can be converted from all dose distributions to C-linac dose distributions.

## Data Availability

The research data were stored in an institutional repository and shared upon request to the corresponding author.
